# Through thick and thin: dual regulation of insect flight muscle and cardiac muscle compared

**DOI:** 10.1007/s10974-019-09536-8

**Published:** 2019-07-10

**Authors:** Belinda Bullard, Annalisa Pastore

**Affiliations:** 1 Department of Biology, University of York, York, YO10 5DD UK; 2 The Wohl Institute, King’s College London, 5 Cutcombe Road, London, SE5 9RT UK

**Keywords:** Insect flight muscle, Cardiac muscle, Troponin, Thick filament regulation

## Abstract

Both insect flight muscle and cardiac muscle contract rhythmically, but the way in which repetitive contractions are controlled is different in the two types of muscle. We have compared the flight muscle of the water bug, *Lethocerus*, with cardiac muscle. Both have relatively high resting elasticity and are activated by an increase in sarcomere length or a quick stretch. The larger response of flight muscle is attributed to the highly ordered lattice of thick and thin filaments and to an isoform of troponin C that has no exchangeable Ca^2+^-binding site. The Ca^2+^ sensitivity of cardiac muscle and flight muscle can be manipulated so that cardiac muscle responds to Ca^2+^ like flight muscle, and flight muscle responds like cardiac muscle, showing the malleability of regulation. The interactions of the subunits in flight muscle troponin are described; a model of the complex, using the structure of cardiac troponin as a template, shows an overall similarity of cardiac and flight muscle troponin complexes. The dual regulation by thick and thin filaments in skeletal and cardiac muscle is thought to operate in flight muscle. The structure of inhibited myosin heads folded back on the thick filament in relaxed *Lethocerus* fibres has not been seen in other species and may be an adaptation to the rapid contractions of flight muscle. A scheme for regulation by thick and thin filaments during oscillatory contraction is described. Cardiac and flight muscle have much in common, but the differing mechanical requirements mean that regulation by both thick and thin filaments is adapted to the particular muscle.

## Introduction

**Fig. 1:**
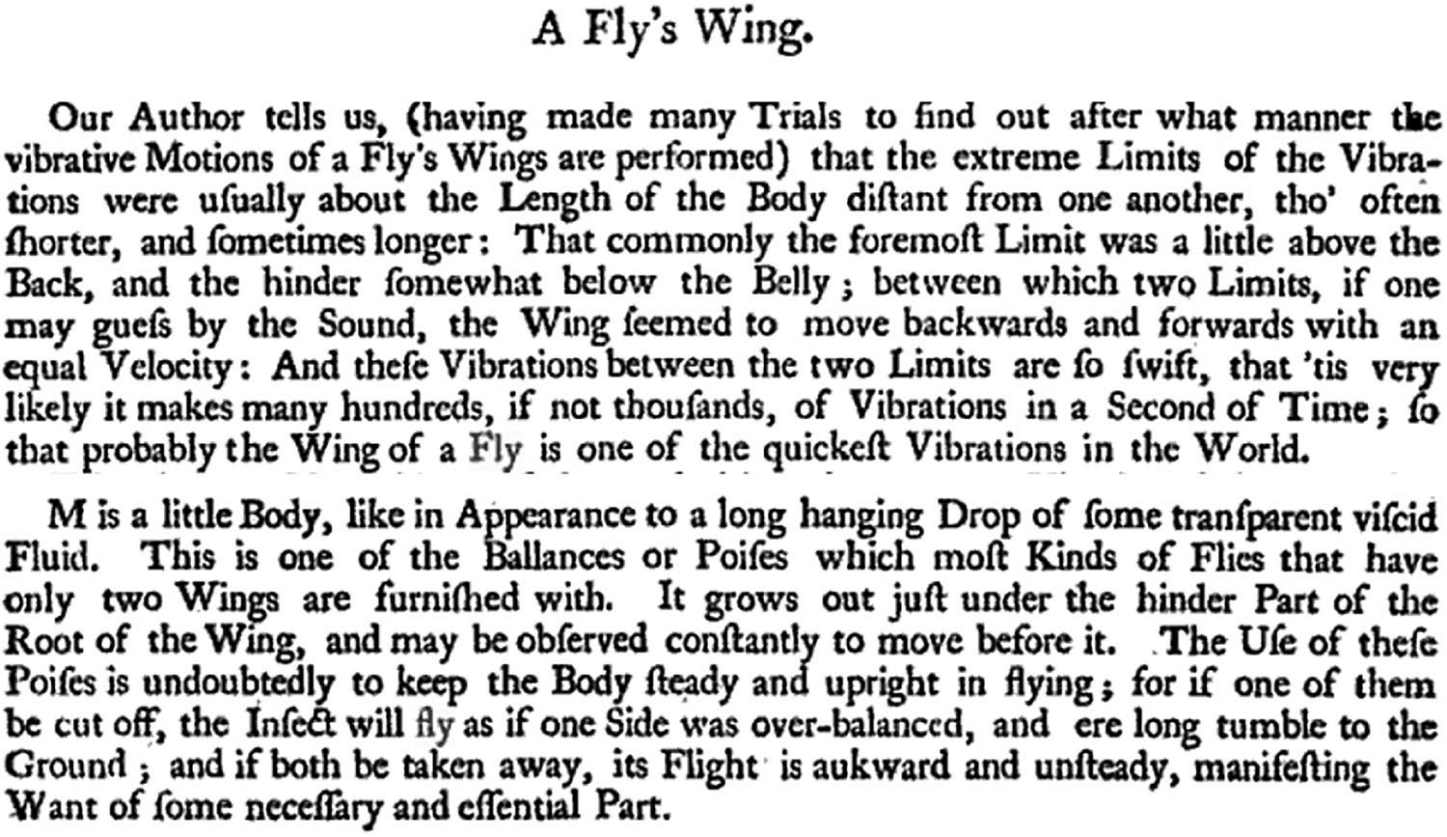
Observations made by Robert Hooke on a fly. This paragraph is taken from a summary of Hooke’s observations published in Micrographia: Or, Some Physiological Descriptions of Minute Bodies Made by Magnifying Glasses. With Observations and Inquiries Thereupon (Hooke 1665)

Robert Hooke (1665) observed the high frequency oscillations of the haltere in Diptera and recognised their importance in keeping insects with high wing beat frequency in balance during flight (Fig. 1). He also appreciated that wing beat frequency could be estimated by the sound. The haltere is a modified hindwing in the Diptera. It was nearly 300 years before Pringle (1949) showed that the haltere muscle of another fly (*Calliphora*) is stimulated to contract at 100–150 Hz by resonant oscillations of the thorax, independently of any repetitive input from thoracic nerves. The result with the haltere led Pringle to investigate the flight muscles of *Calliphora*, and he found that the oscillating contractions are also activated by changes in the shape of the thorax (Pringle 1949). The opposing dorsal longitudinal and dorso-ventral muscles are attached directly to the thoracic cuticle; when one muscle contracts, the other experiences a rapid stretch, due to distortions in the shape of the thorax. After a short delay, the stretched muscle contracts during the release phase of the cycle. Stretch-activation (SA) of flight muscle enables the wingbeat frequencies of flies to reach 200 Hz, without rapid changes in Ca^2+^ levels. The majority of flying insects (75%) have indirect flight muscle (IFM) activated by stretching (Josephson et al. 2000). The flight muscles of the giant waterbug, *Lethocerus*, have been used to study the structure and function of IFM. Another early microscopist and contemporary of Hooke’s, the Dutch van Leeuwenhoek (1719), was the first to see branched fibres and banded patterns in heart muscle.

## Dual regulation

IFM and cardiac muscle have much in common. Both perform rhythmic contractions and both are noticeably activated by an increase in sarcomere length (SL), or by a quick stretch. However, the way in which similar outcomes are achieved often differs in the two types of muscle. Each heart beat is activated by a pulse of Ca^2+^ ions, and the muscle relaxes when Ca^2+^ is removed. Although Ca^2+^ is necessary for the oscillatory contractions of IFM, the concentration remains nearly constant as the force fluctuates; the stimulus for contraction is stretch, not Ca^2+^ (Pringle 1978).

The geometry of the filament lattice in IFM is an important factor in the stretch-activation mechanism. Thin filaments are placed midway between two thick filaments, whereas in cardiac and skeletal muscle, each thin filament is equidistant from three thick filaments. Actin in the thin filaments of IFM has a half pitch of 38.7 nm, compared to 36.0 nm in vertebrate actin (Reedy and Reedy 1985; AL-Khayat et al. 2003). The longer half pitch in IFM is not an intrinsic property of the actin, but is likely to be due to the constraining influence of other thin filament proteins (Ruiz et al. 1998). The periodicity of troponin (Tn) on the thin filament is also 38.7 nm and all Tns on the filament have the same azimuthal orientation relative to the two neighbouring thick filaments. This is not the case for skeletal and cardiac muscle, where the 38.7 nm periodicity of the Tn does not match the half pitch of actin in the thin filament. In IFM, there are target zones every 38.7 nm on the thin filament that are favourably placed for interaction with myosin heads projecting from the thick filament (Tregear et al. 2004; Wu et al. 2010). The target zones are two actin monomers on each side of the long pitch actin helix; they are midway between the Tns so that targets and Tns alternate on the thin filament (Schmitz et al. 1994; Reedy et al. 1994; Wu et al. 2010; Bullard and Pastore 2011).

Contraction of striated muscle is under dual control by the thin filament and the thick filament. According to the steric blocking model, in relaxed skeletal and cardiac muscles, tropomyosin (Tm) on the thin filament is held in a blocking position on actin by Tn, which prevents binding of myosin crossbridges. Troponin has three subunits: TnT, binds the complex to tropomyosin, TnI binds to TnT and to the Ca^2+^ binding subunit, TnC; TnI also binds to actin in relaxing conditions. When Ca^2+^ binds to TnC in the active muscle, TnI is released from actin, and tropomyosin shifts so that myosin binding sites are partially exposed. Myosin binds co-operatively to the thin filament, the binding sites become fully open and the muscle contracts (McKillop and Geeves 1993; Gordon et al. 2000). Recent work has shown that in relaxed muscle, myosin heads bind to each other and to the thick filament backbone in an inhibited head motif (IHM) that maintains the filaments in an OFF state (Woodhead et al. 2005; Alamo et al. 2008; Zoghbi et al. 2008; Sulbaran et al. 2013; Hu et al. 2016). The OFF state is characterised by low ATPase activity and is also known as the super relaxed (SRX) state (Stewart et al. 2010). The majority of myosin heads are inhibited from binding to actin until thick filaments experience a force and heads in the IHM are released to the ON state; the number of heads turned ON is proportional to the force (Reconditi et al. 2014; Linari et al. 2015; Fusi et al. 2016; Irving 2017; Reconditi et al. 2017).

## Length-dependent activation and stretch-activation

IFM has greater passive tension than cardiac muscle and both are stiffer than skeletal muscle (Kulke et al. 2001; Neagoe et al. 2003; Miller et al. 2004; Linke 2007; Fukuda et al. 2008). Passive tension is due to proteins containing tandem Ig-domains that span the I-band, linking the ends of thick filaments to the Z-disc (Labeit et al. 1992; Trinick 1996); Houmeida et al. 2008). IFM fibres shorten by only about 3% during oscillatory contractions and the sarcomeres have exceptionally short I-bands. Kettin, which is largely responsible for passive tension in IFM, has tandem Ig-domains but no extensible sequence; it is attached to the ends of thick filaments, but extends no further along the filaments (Kolmerer et al. 2000; Kulke et al. 2001; Leake et al. 2003; Bullard et al. 2005). Titin in cardiac and skeletal muscles spans the half sarcomere from the Z-disc to the M-line and in the A-band it is bound to the thick filament. The I-band region of cardiac titin has fewer Ig-domains and less extensible sequence than the skeletal isoforms (Cazorla et al. 2000; Neagoe et al. 2003). A relatively inextensible sarcomere means force is rapidly transmitted to the thick and thin filaments when the fibre is stretched. Kettin appears to have the sole function of maintaining the high stiffness of IFM, whereas titin has multiple functions. Cardiac titin can have varying stiffness, depending on the demands of the heart; phosphorylation and signalling through ligands bound to titin domains adapt the molecule to conditions (Krüger and Linke 2011; Linke 2018). Titin also determines the length of the thick filament (Tonino et al. 2017). *Lethocerus* IFM thick filaments have a core of paramyosin, which would reduce the compliance of the filaments and contribute to the greater stiffness of the sarcomere compared to cardiac muscle.

**Fig. 2:**
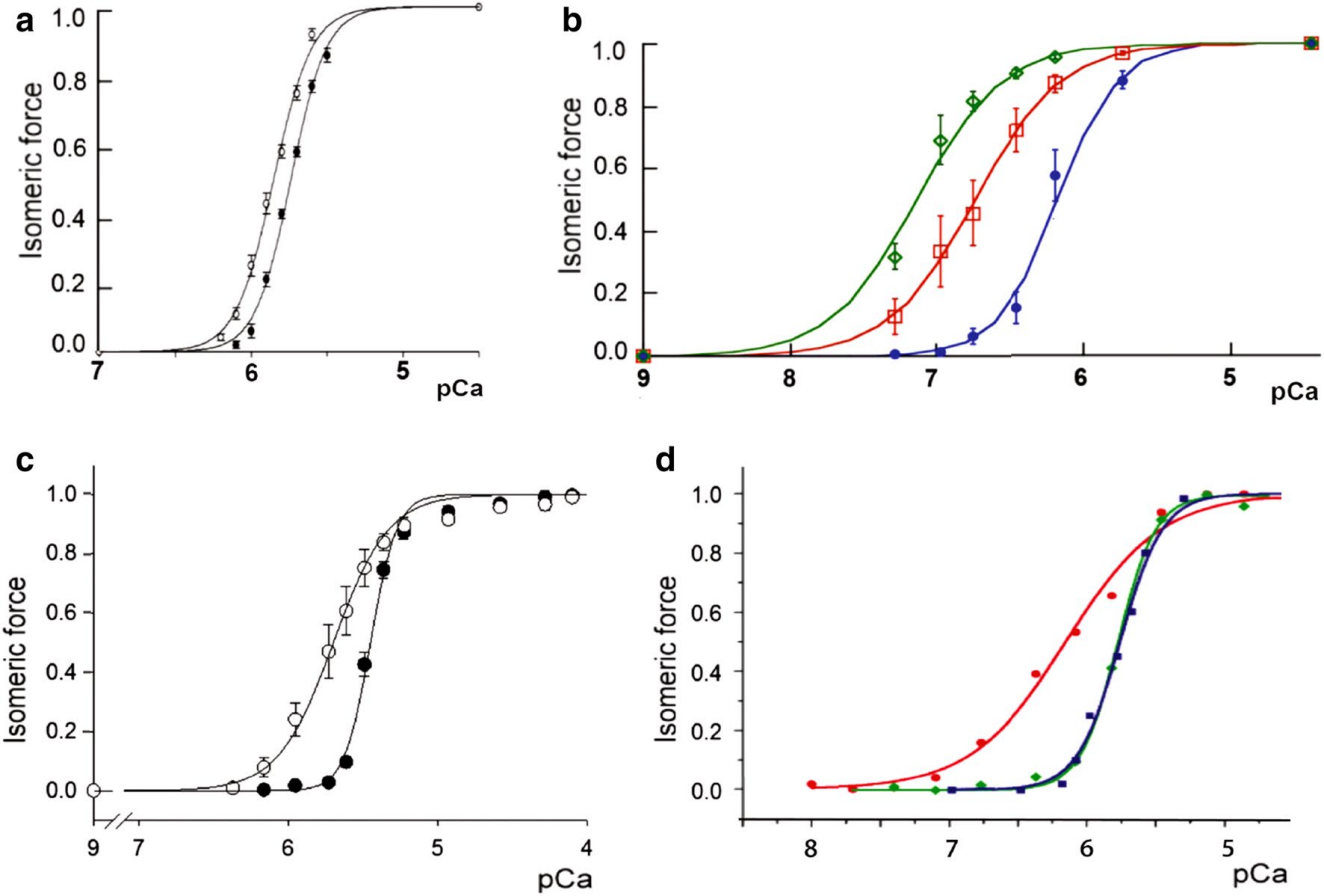
Changes in the Ca^2+^ sensitivity of cardiac fibres and *Lethocerus* IFM. **a** and **b** The effect of SL. **a** Mouse ventricular myocardium at SL: filled black circles 1.9 µm, pCa_50_ 5.7, n_H_ 3.9; open circles 2.25 µm, pCa_50_ 5.8, n_H_ 3.6. From Fig. 1 in Stelzer and Moss (2006). **b***Lethocerus* fibres at SL: filled blue circles 2.5 µm, pCa_50_ 6.2, n_H_ 2.1; open red squares 2.53 µm, pCa_50_ 6.8, n_H_ 1.5; open green diamonds 2.56 µm, pCa_50_ 7.2, n_H_ 1.5. Mean ± SD, n = 3–6 from 1–3 fibres. From R.J. Edwards and M. K. Reedy (unpublished). pCa scales in **a** and **b** are the same. **c** and **d** The effect of MyBP-C or TnC. **c** Rat ventricular trabeculae: filled black circles with no additions pCa_50_ 5.5, n_H_ 5.0; open circles with added MyBP-C fragment C1mC2 (2 µM) pCa_50_ 5.7, n_H_ 2.4. From Fig. 4 in Kampourakis et al. (2014). **d***Lethocerus* IFM: filled red circles native fibres pCa_50_ 6.2, n_H_1.3; filled green diamonds IFM in which TnC is replaced with TnC-F2 pCa_50_ 5.8, n_H_ 3.2; filled blue squares mouse ventricular myocardium SL 2.25 µm, pCa_50_ 5.8, n_H_ 3.6. *Lethocerus* results are from Bullard and Leonard (unpublished), mouse result is from Fig. 1 in Stelzer and Moss (2006). pCa scales are the same in **c** and **d**

Length-dependent activation (LDA) underlies the Frank-Starling mechanism in which the force developed by the ventricular muscle of the heart during systole increases at longer sarcomere lengths; the sarcomere length depends on the extent of ventricular filling during diastole (de Tombe et al. 2010). A stiff titin is essential for LDA (Ait-Mou et al. 2016). The increased force is due to a greater sensitivity of the contractile elements to Ca^2+^. In Fig. 2, isometric force developed by ventricular myocardium and IFM with increasing Ca^2+^ concentrations at different sarcomere lengths is compared. Cardiac muscle produces a relatively small increase in force when sarcomere length is increased by as much as 18%; the Ca^2+^ sensitivity is only increased by 0.1 pCa_50_ unit (Stelzer and Moss 2006) (Fig. 2a). For IFM, a much smaller increase in sarcomere length of 2% results in a pCa_50_ increase of about 1.0 unit (Fig. 2b). The cooperativity of the dependence of force on Ca^2+^ concentration is greater in ventricular myocardium than in IFM, but in both cases, there is little change when the sarcomere length is increased. Both cardiac muscle and IFM have the potential to be activated above the level attained by fibres at rest length, either by an increase in sarcomere length, or by an increase in Ca^2+^ concentration. Clearly, the effect of sarcomere length is greater in IFM. LDA enables the ventricle to produce force during systole that varies depending on the filling of the ventricle during diastole. The effect of increased sarcomere length during diastole on systolic force is independent of the effect of load on the activation of thick filaments (Reconditi et al. 2017; Piazzesi et al. 2018).

Stretch activation (SA) and LDA both produce additional force, but probably by different mechanisms. A rapid stretch applied to a muscle that is already partially activated by Ca^2+^, results in a delayed rise in force; as the muscle shortens on release and the force decreases, relaxation is enhanced by a delayed shortening deactivation. At any particular muscle length, the force is greater during the shortening than during the lengthening phase, which produces a net output of work. SA is probably of minor importance in cardiac muscle compared to IFM. SA of isolated ventricular myocardium is greater at longer sarcomere lengths and it is suggested that when the myocardium is stretched during diastole, SA increases systolic force and delays relaxation. This would increase the volume of blood ejected at each heartbeat and is thought to increase the steepness of the Frank-Starling effect (Stelzer and Moss 2006; Campbell and Chandra 2006). The SA effect is relatively small: the maximum increase in delayed force after stretching isolated ventricular myocardium is about half the pre-stretch force (Stelzer et al. 2006a). This compares with a maximum SA of 3–5 times the pre-stretch force in *Lethocerus* IFM (Agianian et al. 2004; Linari et al. 2004).

## Modulating regulation

Myosin-binding protein C (MyBP-C) affects the activation state of both thick and thin filaments in skeletal and cardiac muscle. The C-terminal region of MyBP-C is bound to the shaft of the thick filament; the N-terminal region extends from the thick filament and can bind either to the thin filament or to the S2 region of myosin. The interactions of the N-terminal region depend on the level of phosphorylation (Pfuhl and Gautel, 2012; Craig et al. 2014). At low concentrations of Ca^2+^, N-terminal MyBP-C partially, or fully, activates the thin filament and inhibits the thick filament (Craig et al. 2014; Kampourakis et al. 2014; Mun et al. 2014; Harris et al. 2016). The effect of cardiac MyBP-C on myosin heads in the thick filaments of cardiomyocytes has been determined by measuring the proportion of heads in the SRX state when MyBP-C is removed in homozygous knockout mice (McNamara et al. 2016). The decrease in the proportion of heads in the SRX state is consistent with increased disorder in the heads and disruption of the IHM in the knockout mice (Zoghbi et al. 2008). It is therefore likely that MyBP-C stabilises the IHM in the OFF state of the filament (Kampourakis et al. 2014; Irving 2017). As myosin heads bind to actin, MyBP-C may be displaced from the IHM and thick filaments turned ON. It has also been suggested that MyBP-C could be a stress sensor that disrupts the IHM in the OFF state of the thick filament (Harris 2016; Irving 2017; Piazzesi et al. 2018). The effect of a low concentration N-terminal MyBP-C on the force developed by cardiomyocytes or trabeculae is to increase the Ca^2+^-sensitivity, so that the force pCa curve is shifted to lower Ca^2+^ concentrations; the cooperativity of force development is decreased (Fig. 2c) (Kampourakis et al. 2014; Harris et al. 2016). The function of MyBP-C may be to modulate the response of cardiac muscle to activation, effectively allowing a graded response to Ca^2+^ and an external force.

The force developed by isolated *Lethocerus* IFM fibres has high Ca^2+^ sensitivity and low cooperativity, with a force-pCa curve similar to that of cardiac muscle with added N-terminal MyBP-C (Fig. 2d). IFM does not have MyBP-C and the characteristic dependence of force on Ca^2+^ concentration is due to the troponin complex in the flight muscle, which differs from the complex in skeletal and cardiac muscles (Bullard and Pastore 2011). The troponin has the subunits, TnT, TnH (TnI) and TnC. TnH has the sequence of TnI in the N-terminal half and a proline-alanine-rich sequence in the C-terminal half. There are two isoforms of TnC: the major isoform, TnC-F1, has a single Ca^2+^-binding site in the C-lobe and no exchangeable Ca^2+^ site in the N-lobe; a minor isoform, TnC-F2 has one Ca^2+^-binding site in the C-lobe and an exchangeable Ca^2+^ site in the N-lobe. TnC-F1 is essential for stretch-activated contractions, whereas TnC-F2 promotes Ca^2+^-activated isometric contraction (Agianian et al. 2004; Krzic et al. 2010). The high Ca^2+^ sensitivity and low cooperativity of the IFM force-pCa curve is due to TnC-F1. When the TnC in fibres is replaced with TnC-F2 alone, the curve is the same as that of cardiac fibres that have been activated by stretching (Fig. 2d). Like TnC-F2, cardiac TnC (cTnC) has only one exchangeable Ca^2+^ site in the N-lobe, (compared to the two exchangeable sites in skeletal TnC). Therefore, the force developed by IFM is restrained from cardiac-like Ca^2+^ dependence by a TnC that has no exchangeable Ca^2+^. The dominance of the effect of TnC-F1 over TnC-F2 in native fibres is likely to be due to the 9:1 stoichiometry. The high sensitivity of IFM to Ca^2+^ means that stretch-activated oscillatory contractions can occur at priming concentrations of Ca^2+^, where there is little isometric force. IFM and cardiac muscle bring about a similar change in Ca^2+^ sensitivity by different means.

Phosphorylation of myosin light chains modulates contraction in vertebrate skeletal and cardiac muscle and in insect flight muscle. The myosins have essential (ELC) and regulatory (RLC) light chains. Phosphorylation of the RLC in skeletal and cardiac muscles increases force at low concentration of Ca^2+^ and this is associated with an increase in disorder of myosin heads (Levine et al. 1996; Stelzer et al. 2006b; Stull et al. 2011; Kampourakis et al. 2016). The change in Ca^2+^ sensitivity is comparable to that obtained by an increase in sarcomere length, suggesting both RLC phosphorylation and LDA act in the same way to produce a more ON state of the thick filament.

The RLC of IFM myosin has an extended sequence at the N-terminus, similar to that at the N-terminus of cardiac myosin ELC. The cardiac ELC extension can bind to actin and is thought to maintain the optimal position of the myosin head for production of force (Trayer et al. 1987; Lowey et al. 2007). The function of the RLC in IFM myosin has been investigated in living *Drosophila* and in isolated fibres (Tohtong et al. 1995; Dickinson et al. 1997; Moore et al. 2000; Farman et al. 2009). Mutant flies lacked the two phosphorylation sites in the RLC, or had the N-terminal extension truncated. In both cases, X-ray diffraction of the IFM showed decreased myosin mass associated with thin filaments, and myosin heads were more disordered in the phosphorylation mutants. The N-terminal extension of the RLC binds to actin and may align myosin heads with the target zones. Mechanics of the isolated IFM suggests that fewer crossbridges are recruited in phosphorylation mutants and electron micrographs of Ca^2+^-activated fibres are consistent with this. As in skeletal and cardiac muscle, phosphorylation of the RLC in IFM may increase the ON state of the thick filament, resulting in more available myosin heads. However, there was no significant effect on the Ca^2+^ sensitivity of isometric force developed by mutant IFM fibres (Dickinson et al. 1997). Mutant *Drosophila* could fly. The power output was reduced, but this was partially compensated for by an increase in wingbeat frequency and in the amplitude of the stroke. Therefore, RLC phosphorylation and an RLC extension are modulating factors, rather than being essential for flight.

## *Lethocerus* TnC and its interactions with TnH and TnI

TnC-F1 and F2 share 47% sequence identity and thus must have a similar fold. They could not be crystallized and were studied by solution nuclear magnetic resonance (NMR). Ca^2+^ binds in EF-hand 4 in TnC-F1 and in EF-hands 2 and 4 in TnC-F2 (Agianian et al. 2004). Their structure consists in a typical EF-hand fold with two globular domains spaced by a flexible linker. Each domain contains two EF-hand motifs, each of which comprises two α-helices that flank an eleven residues loop, which is where Ca^2+^ binds when the loop contains six canonical residues. The two loops form the typical short antiparallel β-sheet that keeps the two EF-hands side by side. A short N helix precedes the A helix, but it is formed only in some of the NMR structures of TnC-F1 (De Nicola et al. 2007). As in all calmodulin-like folds, the two lobes are connected by a tethering helix which in some structures is a straight helix that confers a dumbbell shape to the protein (Herzberg and James 1985; Houdusse et al. 1997; Satyshur et al. 1988). In other X-ray and solution structures, the linker is disordered and unstructured, such as in TnC-F1 and TnC-F2 and cardiac TnC. This arrangement allows an undetermined relative orientation of the two lobes that can be instrumental in favouring interactions (Blumenschein et al. 2005; Dvoretsky et al. 2002; Slupsky and Sykes 1995; Sia et al. 1997; Vinogradova et al. 2005). The arrangement of TnC-F1 and -F2 is different and parallels the Ca^2+^-binding capacity of the two proteins. The N lobe of TnC-F1 is in a closed conformation and has closest resemblance with the structure of the apo N lobe of skeletal TnC (1skt, Tsuda et al. 1999). Conversely, the holo C lobe of TnC-F1 shares highest similarity with the holo C lobe of human cardiac TnC in a complex with the inhibitory region of TnI (1ozs, Lindhout and Sykes, 2003). This implies that this lobe is in an open state and indicates that a single Ca^2+^ ion is sufficient to induce opening of the domain and that, already in its Ca^2+^-bound state, it is prepared to interact with TnH. This is unlike EF hand 2 in the N-lobe of cardiac TnC, which also binds a single Ca^2+^, but is only in an open state when in complex with the switch region of TnI.

For comparison, both apo and holo forms of TnC-F2 are also structured in the absence of Ca^2+^ and have a comparable helical content. The role of Ca^2+^ is thus not structural. This is in contrast to the behaviour of the C lobe of skeletal and cardiac TnC. This lobe is unstructured in the absence of Ca^2+^ and acquires structure only in the presence of the cation (De Nicola et al. 2010). The dynamic properties of TnC-F1 and -F2 are overall similar (Sanfelice et al. 2016; De Nicola et al. 2007).

TnC-F1 and -F2 interact with TnH. TnC-F1 binds the TnH(30-61) peptide with a K_d_ of 1.9 nM and 0.9 nM in the absence and presence of Ca^2+^ respectively. TnH(30-61) binds TnC-F2 with a K_d_ of 8 nM in the presence of Ca^2+^ and 40 nM in the absence of Ca^2+^, or in the presence of the more weakly coordinated magnesium (Martin et al. 2011). The affinities of the TnH(126–159) peptide for TnC-F1 and TnC-F2 are comparable and around 13 µM and 3 µM in the absence and presence of Ca^2+^. Two-hybrid screening and NMR studies demonstrated that the N-terminus of TnH (residues 1–76) and the C-terminal lobe of TnC-F1 are necessary and sufficient for interaction of the two molecules (De Nicola et al. 2007). The N lobe of TnC-F1 does not bind to TnH. Consistent with these results, the NMR spectra of TnC-F1 in complex with TnH(30–61), or with the longer fragments TnH(1–224) and TnH(1–340)) are similar, apart from the latter being broader because of the slower tumbling. This indicates that the region 30–61 of TnH is key for the interaction with TnC-F1. Likewise, TnC-F2 interacts with the peptides TnH(30–61) and TnH(126–159), designed by homology with regions involved in the interaction in vertebrates (De Nicola et al. 2007). The C lobe binds primarily TnH(30–61), whereas the N lobe interacts with TnH(126–159). This behaviour is similar to that observed in vertebrate troponin complexes.

## Experimentally based models of the *Lethocerus* Tn heterotrimer

**Fig. 3:**
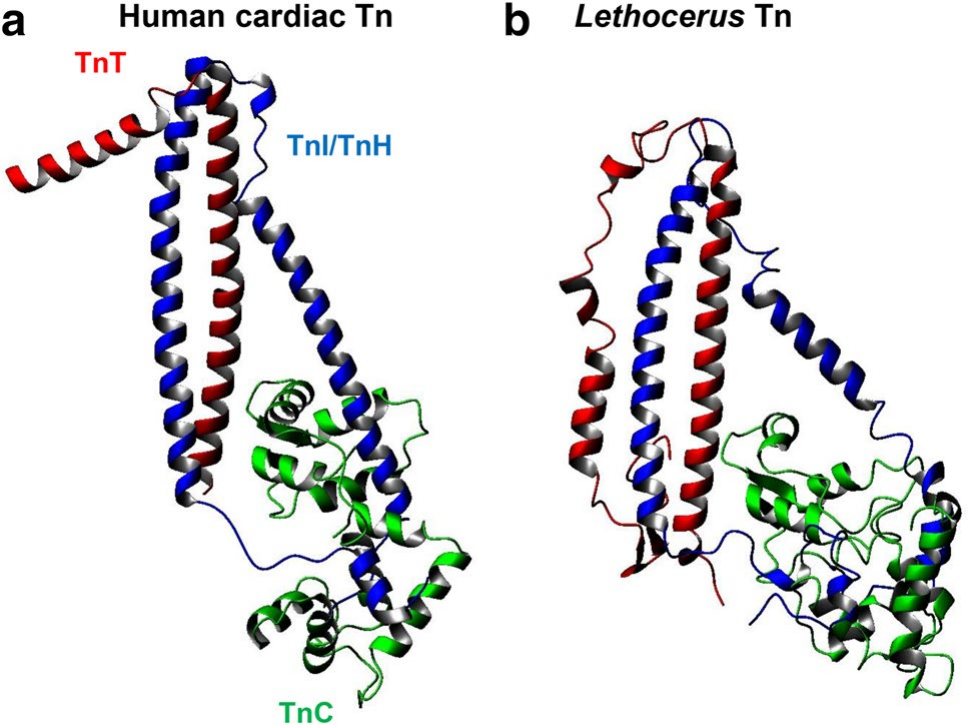
Comparison of the X-ray structures of the human cardiac and *Lethocerus* IFM Tn complexes. **a** Human cardiac Tn (1J1D). **b***Lethocerus* model of the Tn complex with TnC-F2, generated by homology, using cardiac Tn as a template. *Lethocerus* TnC and TnH/TnI do not have major insertions or deletions; TnT however has three insertions in the *Lethocerus* Tn structure. The complex was modelled by an iterative gap-growing process in which residues were gradually added. After each insertion, the system was relaxed by molecular dynamics runs (Sanfelice et al. 2016). The degree of homology between TnC-F1 and TnC-F2 is so high that the Tn structures will be similar at this level of resolution

Modelling the structure of the *Lethocerus* Tn complex with TnC-F2 was attempted using the coordinates of the vertebrate cardiac complex (PDB ID: 1J1D) as template (De Nicola et al. 2007; Sanfelice et al. 2016). The model obtained recapitulates all the important features of the complex and provides an excellent starting structure for further studies (Fig. 3). The main problem encountered was found in TnT. *Lethocerus* TnC and TnI do not have major insertions or deletions compared to the vertebrate sequences. Standard homology modelling could thus be used to build their structures. In contrast, *Lethocerus* TnT has three large insertions, one in the middle of the first helix of the hairpin. The inserted residues were thus introduced by an iterative gap growing process alternated with a relaxation phase in a molecular dynamics calculation. In this way, the peptide chain could be lengthened progressively while maintaining the structure of the trimer in an energetically favourable state compatible with the template. The resulting TnT model suggests the presence of an insertion in the *Lethocerus* sequence which ends up with the hairpin loop. As a result, the overall shape of the complex remains similar but the orientation of the N-terminal helix of TnT differs from that of the template, while keeping the pattern of intermolecular interactions with TnI and TnC unaltered (De Nicola et al. 2007). The similarity in the force-pCa curves for *Lethocerus* fibres substituted with TnC-F2 and cardiac fibres with cTnC (Fig. 2d), is consistent with the similarity in the structures of *Lethocerus* Tn with TnC-F2 and cardiac Tn (Fig. 3).

## Common features in regulation by TnC-F1 and cardiac TnC

**Fig. 4:**
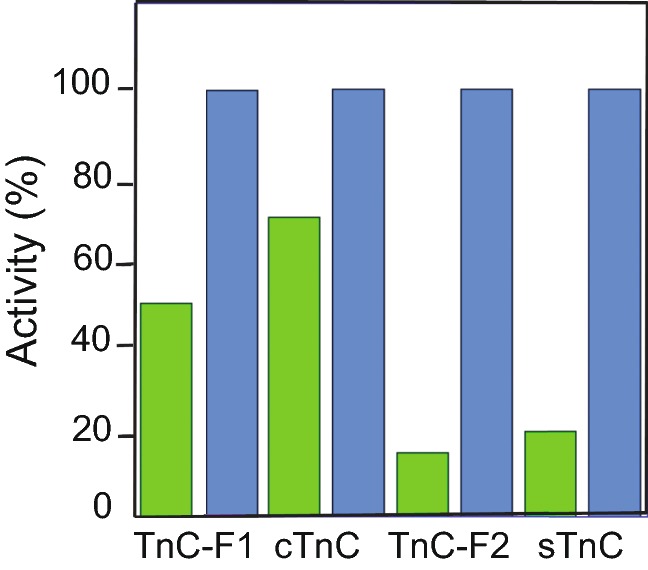
Activation of cardiac and IFM thin filaments by TnC. The extent of activation of thin filaments by Ca^2+^ and by myosin S1 was estimated in solution (see text). Values are normalised to the maximum level of activation. IFM thin filaments have actin and *Lethocerus* Tm-Tn with TnC-F1 or TnC-F2. Cardiac thin filaments were isolated from pig hearts. Skeletal thin filaments were reconstituted from actin, Tm and Tn. Myosin S1 was from rabbit skeletal muscle. Green bars are the proportion of filaments in the open state at pCa 4.0–4.5; blue bars represent the maximum activity produced by Ca^2+^ and myosin S1. Thin filaments with *Lethocerus* TnC-F1 or TnC-F2 (Boussouf et al.^2007^); with cardiac TnC (cTnC) (Risi et al. 2017); with skeletal TnC (sTnC) (Heeley et al. 2006)

There are similarities between TnC-F1 and cTnC in how they affect the thin filament at a *maximal* concentration of Ca^2+^ (pCa 4.0–4.5). Equilibrium binding measurements with myosin S1 and regulated actin in Geeves’s laboratory, using thin filaments with the *Lethocerus* Tm-Tn complex and TnC-F1, showed that thin filaments regulated by TnC-F1 were about equally in the open and closed states at pCa 4.5 (Boussouf et al. 2007). This is in contrast to the effect of the troponin complex with TnC-F2, where filaments were 15% open and 78% closed at pCa 4.5. Recently, the activation state of cardiac thin filaments has been estimated by stopped-flow kinetic measurements of Pi release from the thin filament-myosin-ADP-Pi complex. Cardiac thin filaments were activated by Ca^2+^ to ~ 70% of the maximum level, without binding myosin heads (Risi et al. 2017). The activation of IFM, cardiac and skeletal thin filaments by TnC are compared in Fig. 4. TnC-F1 and cTnC activate thin filaments to a 50–70% open state in the presence of Ca^2+^, although TnC-F1 has no exchangeable Ca^2+^ in the N-lobe, while cTnC has one. Activation of IFM filaments by TnC-F2 is similar to that of skeletal TnC (sTnC). In the case of IFM, activation at this high concentration of Ca^2+^ would not be physiological because in the native fibre, TnC-F2 would dominate the effect of TnC-F1. As discussed above, the structures of IFM and vertebrate Tn complexes are similar, although there are differences in the sequences of the subunits.

## Thick filament regulation

Both cardiac and IFM thick filaments are regulated by the force applied to the filaments, although the structure and protein composition of the two are different. In cardiac and skeletal muscle thick filaments, three myosin heads emerge from the filament every 14.3 nm. The spacing of these crowns changes from 14.3 nm in relaxed fibres to 14.5 nm on activation (Haselgrove 1975; Linari et al. 2015). In *Lethocerus* thick filaments, there are four myosin heads per crown (Reedy et al. 1981; Morris et al. 1991) and the spacing of crowns is 14.5 nm in both relaxed and activated fibres (Miller and Tregear 1972; Perz-Edwards et al. 2011).

The form of the IHM in relaxed fibres is similar in the striated muscle of several species and in cardiac muscle (Woodhead et al. 2005; Zoghbi et al. 2008; Al-Khayat et al. 2013; Alamo et al. 2008; Sulbaran et al. 2013). Within a myosin molecule, a ‘blocked’ head binds to a ‘free’ head, which prevents the blocked head from binding to the thin filament, but leaves the actin-binding site of the free head available; the blocked head also contacts the S2 region of the same molecule and that of an adjacent molecule. The IHM in these muscles lies on the surface of the thick filament, forming a characteristic J shape. Activation of myosin heads on the thick filament is adjusted to the load on the filaments. X-ray diffraction measurements on cardiac trabeculae have shown that during diastole, the thick filament is in the OFF state, and during systole only some of the myosin heads are in the ON state; the proportion varies with the load on the filament, which is determined by the arterial pressure (Reconditi et al. 2017; Piazzesi et al. 2018). When cardiac muscle is activated, the change in the axial periodicity of the myosin heads from 14.3 to 14.5 nm is associated with a loss of helical order in the filament backbone. The conversion of myosin heads from the OFF state in the IHM to the ON state may be due to disruption of stabilising interactions with MyBP-C, as discussed above, However, there is not enough MyBP-C for a one-to-one interaction with myosin.

The IHM in *Lethocerus* IFM is unlike that in other muscles. Recently Taylor and colleagues (Hu et al. 2016) have determined the structure of the relaxed thick filament by cryo-electron microscopy. The shafts of myosin molecules in the filament backbone, as well as the position of additional proteins in the thick filament, have been resolved to 5.5–10 Å. Candidates for the additional proteins are myofilin, which is not exposed to the surface of the filament, and flightin, which is on the surface (Ferguson et al. 1994; Qui et al. 2005). These additional proteins are not present in cardiac thick filaments. The structure of the myosin heads in the IHM has a resolution of 12–21 Å, which exceeds the current resolution of the IHM in skeletal and cardiac muscle. Taylor and colleagues (Hu et al. 2016) point out that a resolution of ~ 25 Å or more is needed to determine the structure of the IHM with certainty.

**Fig. 5:**
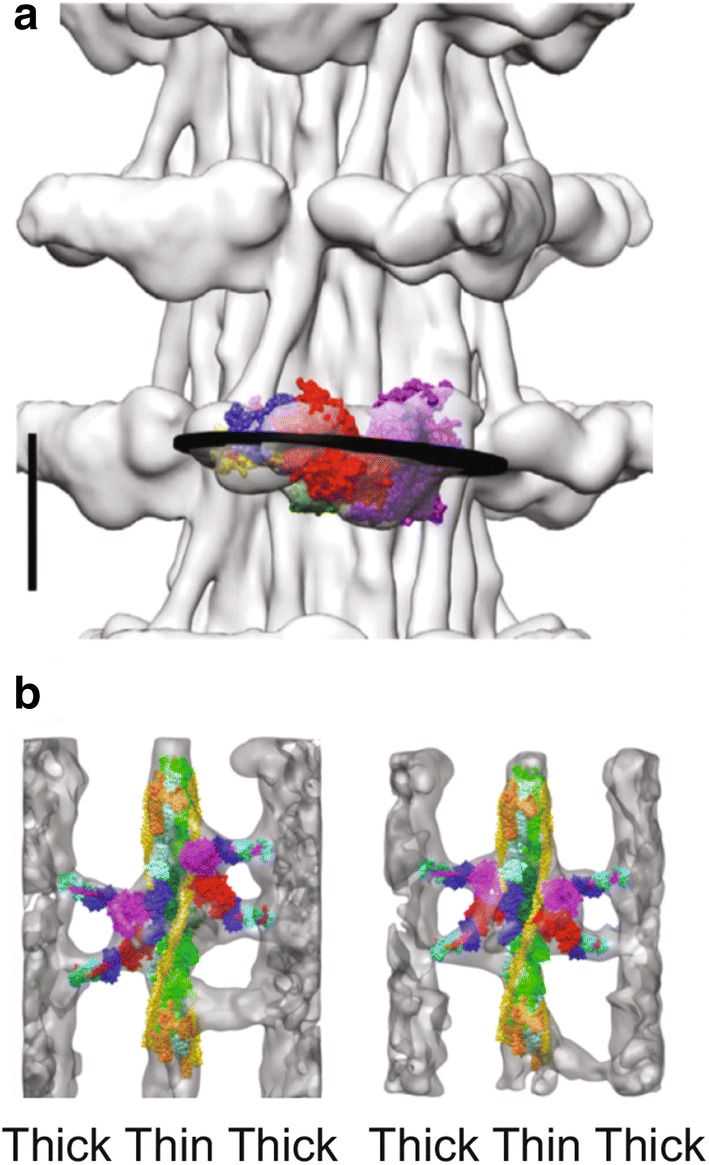
The *Lethocerus* thick filament and troponin bridges. **a** Cryo-EM image of a relaxed IFM thick filament. Myosin heads form crowns spaced at 14.5 nm along the filament. The IHM is nearly perpendicular to the filament axis, with the blocked head projecting furthest from the filament. The bare zone of the filament is at the top. Scale bar 10.0 nm. **b** Averaged images of IFM during isometric contraction. Crossbridges are fitted with atomic models of a myosin head and Tn bridges are not. Tn is orange and Tm is yellow. Tn bridges contact the thin filament at or near Tn. **a** is from Hu et al. (2017); **b** is from Wu et al. (2010)

In the *Lethocerus* IHM, the blocked head interacts with the free head, but not S2, and the free head interacts with the thick filament backbone (Hu et al. 2016; Hu et al. 2017; Taylor et al. 2019). The IHM is perpendicular to the filament axis, and IHMs form the prominent crowns seen at 14.5 nm spacing in relaxed thick filaments (Fig. 5a). The blocked head projects further from the thick filament than the free head. X-ray diffraction measurements have shown that when fibres are stretched, the helical angle between crowns on the thick filament decreases slightly (Perz-Edwards et al. 2011) and there is indirect evidence that disorder in the blocked head, seen by cryo-electron microscopy, is coupled to the change in helical angle in the filament backbone (Hu et al. 2017).

The form of myosin heads in the OFF state of thick filaments, together with X-ray diffraction measurements on fibres during oscillating contractions, has led to a suggestion of how stretch activation might work. Taylor and colleagues (Hu et al. 2017) propose that a rapid stretch might produce disorder in the blocked head, dislodging it from the IHM so that it could bind to a target zone on the thin filament. The blocked head could perform a power stroke, which would pull the free head from the thick filament backbone. Then the free head would bind to a second actin in the same target zone as the blocked head, and perform a power stroke. If there were no other available target zone, the free head would go back to bind to the thick filament backbone, thus limiting the mobility of the blocked head, which would join its partner in an IHM. The disordered blocked head might be a sensor for the state of the thin filament, which would depend on the concentration of Ca^2+^ in the fibres. Based on the ATPase activity of the isolated myosin, the authors calculate that in *Lethocerus* IFM, the myosin heads could cycle from IHM to active crossbridges at the oscillation frequency of the muscle, which is 30-40 Hz in vivo. At present this sequence of events is speculative.

## Troponin bridges

Myosin heads bind reversibly to regulated thin filaments. In skeletal and cardiac muscle, binding sites on actin become available when troponin binds Ca^2+^ and tropomyosin moves from a blocking position on actin. Thin filament activation in these muscles is thought to precede activation of the thick filaments (Irving 2017; Piazzesi et al. 2018). Time-resolved X-ray diffraction measurements of *Lethocerus* IFM during oscillatory contractions at constant Ca^2+^ concentration showed changes in the Tm reflection indicating Tm movement in response to stretch, followed by crossbridge binding and development of force; the changes were reversed when the fibres were released (Perz-Edwards et al. 2011). There was evidence for permanent bridges outside the target zone, at the position of Tn; these had been seen previously in electron micrographs of contracting muscle and called Tn bridges (Fig. 5b) (Wu et al. 2010). It was suggested that Tn bridges pull tropomyosin from a blocking position on actin when the muscle is stretched every contraction cycle, and that tropomyosin resumes the blocking position when the muscle is released. The new findings on thick filament regulation in IFM mean that the effect of stretch on the IHM and on Tn bridges must be co-ordinated at contraction frequencies of up to 40 Hz in *Lethocerus* and 200 Hz in *Drosophila*. This will depend on the compliance of the thick filament backbone as well as that of Tm and Tn bridges.

An inconsistency in thin filament regulation of *Lethocerus* IFM now has a possible explanation. X-ray diffraction measurements on fibres, in which the length was sinusoidally cycled at a constant priming concentration of Ca^2+^ (pCa 5.7), showed the intensity of the tropomyosin reflection changing from a maximum at the peak of the stretch to a minimum at the shortest length (where the intensity was equivalent to that of relaxed fibres at pCa 9) (Perz-Edwards et al. 2011). These changes correspond to the transition from the fully open state of the thin filament to the blocked state. Equilibrium and kinetic binding measurements with myosin S1 and thin filaments regulated by *Lethocerus* Tm-Tn and TnC-F1, as described above, showed that the filaments were about 40% in the open state (available for myosin binding) and 60% in the closed state (partially open) at a priming Ca^2+^ concentration (pCa 6) (Boussouf et al. 2007). Therefore, unstretched fibres would be expected to develop tension under these conditions, but they remain relaxed. However, this might be explained if the thick filaments in the fibre diffraction experiments were OFF until stretched. Stress on the Tn bridges (not measurable in the solution studies) would enhance the activation of the thin filaments and promote tropomyosin movement to the blocking position when the stretch was released. The implication is that when thick filaments are activated by stretching at priming Ca^2+^ concentration, they would find a thin filament already available to bind myosin heads. The function of the unusual TnC-F1 in IFM may be to bias the thin filament towards the partially open state, at the relatively low concentrations of Ca^2+^ at which TnC-F2 is not active. Tn bridges would act as a hairline trigger to expose actin target sites after a stretch. It is possible that in cardiac fibres, as in IFM, thin filaments are primed for interaction with myosin heads, once the heads are freed from the IHM by stress on the thick filaments at each heartbeat.

## Conclusions

Although IFM has more in common with cardiac muscle than with skeletal muscle, there are many differences between the two. IFM is restricted by the requirement for nearly constant contraction frequency, which is determined by the resonant properties of the thorax, including the muscles. The structure of the IFM is adapted to this requirement: matching periodicities of actin, Tn and target zones on the thin filament, as well as the arrangement of crowns on the thick filament, favour rapid controlled interaction between myosin and actin. Cardiac muscle, which has no such constraints, has a less precise structure.

The more pronounced LDA and SA of IFM, and the small changes in sarcomere length at which the effects are observed, are likely to be a consequence of the high stiffness and regular structure of the IFM sarcomere. The changes in Ca^2+^ sensitivity and cooperativity produced by MyBP-C have a modulating effect, resulting in some flexibility in the contractile response of cardiac muscle, whereas the same effects produced by TnC-F1 are essential for the function of IFM. At present, it is not clear if MyBP-C and Tn bridges have a comparable function in transmitting force between thick and thin filaments.

The conversion of myosin heads on the thick filament from OFF to ON depends on the force on the filaments. For IFM during oscillatory contractions, the force depends on the load of the wings. This is expected to be constant, so the number of ON bridges would be the same at each contraction. By contrast, the load on cardiac muscle depends on the arterial pressure and as this varies, the number of ON bridges will vary. Perhaps the unusual structure of the IHM in *Lethocerus* IFM is an adaptation to a rapid conversion between states under a constant load.

Thirty years after his experiments with the haltere, Pringle (1978) foresaw the existence of the IHM in thick filaments and Tn bridges in IFM: “[…] the conclusion must be that strain of the myosin filament, produced by stress either through the connecting protein in insect fibrillar muscle or through those cross bridges that are already attached, changes the organization of the myosin filament in some way that makes more bridges available for interaction. What this change of organisation is is completely unknown at the present time, but is likely to be a geometrical effect.”
